# Efficient ancestry and mutation simulation with msprime 1.0

**DOI:** 10.1093/genetics/iyab229

**Published:** 2021-12-13

**Authors:** Franz Baumdicker, Gertjan Bisschop, Daniel Goldstein, Graham Gower, Aaron P Ragsdale, Georgia Tsambos, Sha Zhu, Bjarki Eldon, E Castedo Ellerman, Jared G Galloway, Ariella L Gladstein, Gregor Gorjanc, Bing Guo, Ben Jeffery, Warren W Kretzschumar, Konrad Lohse, Michael Matschiner, Dominic Nelson, Nathaniel S Pope, Consuelo D Quinto-Cortés, Murillo F Rodrigues, Kumar Saunack, Thibaut Sellinger, Kevin Thornton, Hugo van Kemenade, Anthony W Wohns, Yan Wong, Simon Gravel, Andrew D Kern, Jere Koskela, Peter L Ralph, Jerome Kelleher

**Affiliations:** 1 Cluster of Excellence “Controlling Microbes to Fight Infections”, Mathematical and Computational Population Genetics, University of Tübingen, 72076 Tübingen, Germany; 2 Institute of Evolutionary Biology, The University of Edinburgh, Edinburgh EH9 3FL, UK; 3 Khoury College of Computer Sciences, Northeastern University, Boston, MA 02115, USA; 4 Lundbeck GeoGenetics Centre, Globe Institute, University of Copenhagen, 1350 Copenhagen K, Denmark; 5 Department of Integrative Biology, University of Wisconsin–Madison, Madison, WI 53706, USA; 6 Melbourne Integrative Genomics, School of Mathematics and Statistics, University of Melbourne, Parkville, VIC 3010, Australia; 7 Big Data Institute, Li Ka Shing Centre for Health Information and Discovery, University of Oxford, Oxford OX3 7LF, UK; 8 Leibniz Institute for Evolution and Biodiversity Science, Museum für Naturkunde, Berlin 10115, Germany; 9 Fresh Pond Research Institute, Cambridge, MA 02140, USA; 10 Department of Biology, Institute of Ecology and Evolution, University of Oregon, Eugene, OR 97403-5289, USA; 11 Computational Biology Program, Fred Hutchinson Cancer Research Center, Seattle, WA 98102, USA; 12 Department of Genetics, University of North Carolina at Chapel Hill, Chapel Hill, NC 27599-7264, USA; 13 Embark Veterinary, Inc., Boston, MA 02111, USA; 14 The Roslin Institute and Royal (Dick) School of Veterinary Studies, University of Edinburgh, Edinburgh EH25 9RG, UK; 15 Institute for Genome Sciences, University of Maryland School of Medicine, Baltimore, MD 21201, USA; 16 Center for Hematology and Regenerative Medicine, Karolinska Institute, 141 83 Huddinge, Sweden; 17 Natural History Museum, University of Oslo, 0318 Oslo, Norway; 18 Department of Human Genetics, McGill University, Montréal, QC H3A 0C7, Canada; 19 Department of Entomology, Pennsylvania State University, State College, PA 16802, USA; 20 National Laboratory of Genomics for Biodiversity (LANGEBIO), Unit of Advanced Genomics, CINVESTAV, Irapuato, Mexico; 21 IIT Bombay, Powai, Mumbai 400 076, India; 22 Professorship for Population Genetics, Department of Life Science Systems, Technical University of Munich, 85354 Freising, Germany; 23 Department of Ecology and Evolutionary Biology, University of California, Irvine, CA 92697, USA; 24 Broad Institute of MIT and Harvard, Cambridge, MA 02142, USA; 25 Department of Statistics, University of Warwick, Coventry CV4 7AL, UK; 26 Department of Mathematics, University of Oregon, Eugene, OR 97403-5289, USA

**Keywords:** simulation, coalescent, mutations, Ancestral Recombination Graphs

## Abstract

Stochastic simulation is a key tool in population genetics, since the models involved are often analytically intractable and simulation is usually the only way of obtaining ground-truth data to evaluate inferences. Because of this, a large number of specialized simulation programs have been developed, each filling a particular niche, but with largely overlapping functionality and a substantial duplication of effort. Here, we introduce msprime version 1.0, which efficiently implements ancestry and mutation simulations based on the succinct tree sequence data structure and the tskit library. We summarize msprime’s many features, and show that its performance is excellent, often many times faster and more memory efficient than specialized alternatives. These high-performance features have been thoroughly tested and validated, and built using a collaborative, open source development model, which reduces duplication of effort and promotes software quality via community engagement.

## Teaser

Since its introduction in 2016, the msprime simulator has grown in popularity and is now one of the most commonly used tools in population genetics. This article marks the 1.0 release of msprime and summarizes the many features it has accumulated through an open source community development model. Despite its generality, msprime’s performance is excellent—in many cases orders of magnitude faster and more memory efficient than more specialized methods.

## Introduction

The coalescent process (Kingman 1982a, 1982b; Hudson 1983b; Tajima 1983) models the ancestry of a set of sampled genomes, providing a mathematical description of the genealogical tree that relates the samples to one another. It has proved to be a powerful model, and is now central to population genetics (Hudson 1990; Hein *et al.* 2004; Wakeley 2008). The coalescent is an efficient framework for population genetic simulation, because it allows us to simulate the genetic ancestry for a sample from an idealized population model, without explicitly representing the population in memory or stepping through the generations. Indeed, Hudson (1983b) independently derived the coalescent *in order to* efficiently simulate data, and used these simulations to characterize an analytically intractable distribution. This inherent efficiency, and the great utility of simulations for a wide range of purposes, has led to dozens of different tools being developed over the decades (Carvajal-Rodríguez 2008; Liu *et al.* 2008; Arenas 2012; Hoban *et al.* 2012; Yuan *et al.* 2012; Yang *et al.* 2014; Peng *et al.* 2015). 

Two technological developments of recent years, however, pose major challenges to most existing simulation methods. First, fourth-generation sequencing technologies have made complete chromosome-level assemblies possible (Miga *et al.* 2020), and high quality assemblies are now available for many species. Thus, modeling genetic variation data as a series of unlinked non-recombining loci is no longer a reasonable approximation, and we must fully account for recombination. However, while a genealogical tree relating *n* samples in the single-locus coalescent can be simulated in *O*(*n*) time (Hudson 1990), the coalescent with recombination is far more complex, and programs such as Hudson’s classical ms (Hudson 2002) can only simulate short segments under the influence of recombination. The second challenge facing simulation methods is that sample sizes in genetic studies have grown very quickly in recent years, enabled by the precipitous fall in genome sequencing costs. Human datasets like the UK Biobank (Bycroft *et al.* 2018) and gnomAD (Karczewski *et al.* 2020) now consist of hundreds of thousands of genomes and many other datasets on a similar scale are becoming available (Tanjo *et al.* 2021). Classical simulators such as ms and even fast approximate methods such as scrm (Staab *et al.* 2015) simply cannot cope with such a large number of samples.

The msprime simulator (Kelleher *et al.* 2016; Kelleher and Lohse 2020) has greatly increased the scope of coalescent simulations, and it is now straightforward to simulate millions of whole chromosomes for a wide range of organisms. The “succinct tree sequence” data structure (Kelleher *et al.* 2016, 2018, 2019; Wohns *et al.* 2021), originally introduced as part of msprime, makes it possible to store such large simulations in a few gigabytes, several orders of magnitude smaller than commonly used formats. The succinct tree sequence has also led to major advances in forwards-time simulation (Haller *et al.* 2018; Kelleher *et al.* 2018), ancestry inference (Kelleher *et al.* 2019; Wohns *et al.* 2021), and calculation of population genetic statistics (Kelleher *et al.* 2016; Ralph *et al.* 2020). Through a rigorous open-source community development process, msprime has gained a large number of features since its introduction, making it a highly efficient and flexible platform for population genetic simulation. This paper marks the release of msprime 1.0. We provide an overview of its extensive features, demonstrate its performance advantages over alternative software, and discuss opportunities for ongoing open-source community-based development.

The efficiency of coalescent simulations depends crucially on the assumption of neutrality, and it is important to note that there are many situations in which this will be a poor approximation of biological reality (Johri *et al.* 2021). In particular, background selection has been shown to affect genome-wide sequence variation in a wide range of species (Charlesworth *et al.* 1993, 1995; Charlesworth and Jensen 2021). Thus care must be taken to ensure that the results of purely neutral simulations are appropriate for the question and genomic partition under study. A major strength of msprime, however, is that it can be used in conjunction with forwards-time simulators, enabling the simulation of more realistic models than otherwise possible (Haller *et al.* 2018; Kelleher *et al.* 2018).

## Results

In the following sections, we describe the main features of msprime 1.0, focusing on the aspects that are either new for this version, or in which our approach differs significantly from classical methods (summarized in Table 1). Where appropriate, we benchmark msprime against other simulators, but the comparisons are illustrative and not intended to be systematic or exhaustive. Please see Kelleher *et al.* (2016) for a performance comparison of msprime against simulators such as ms, msms, and scrm.

**Table 1:** Major features of msprime 1.0 added since version 0.3.0 (Kelleher *et al.* 2016)

Interface	Separation of ancestry and mutation simulations. Ability to store arbitrary metadata along with simulation results, and automatic recording of provenance information for reproducibility. Jupyter Notebook (Kluyver *et al.* 2016) integration. Rich suite of analytical and visualization methods via the tskit library.
Ancestry	SMC, SMC’, Beta- and Dirac-coalescent, discrete time Wright–Fisher, and selective sweep models. Instantaneous bottlenecks. Discrete or continuous genomic coordinates, arbitrary ploidy, gene conversion. Output full ARG with recombination nodes, ARG likelihood calculations. Record full migration history and census events. Improved performance for large numbers of populations. Integration with forward simulators such as SLiM and fwdpy11 (“recapitation”).
Demography	Improved interface with integrated metadata and referencing populations by name. Import from Newick species tree, *BEAST (Heled and Drummond 2010), and Demes (Gower *et al.* 2022). Numerical methods to compute coalescence rates.
Mutations	JC69, HKY, F84, GTR, BLOSUM62, PAM, infinite alleles, SLiM and general matrix mutation models. Varying rates along the genome, recurrent/back mutations, discrete or continuous genomic coordinates, overlaying multiple layers of mutations, exact times associated with mutations.

### User interface

The majority of simulation packages are controlled either through a command-line interface (*e.g.*, Hudson 2002; Kern and Schrider 2016), a text-based input file format (*e.g.*, Guillaume and Rougemont 2006; Excoffier and Foll 2011; Shlyakhter *et al.* 2014), or a mixture of both. Command-line interfaces make it easy to run simple simulations, but as model complexity and the number of parameters increase, they become difficult to understand and error-prone (Ragsdale *et al.* 2020; Gower *et al.* 2022). Specifying parameters through a text file alleviates this problem to a degree, but lacks flexibility, for example, when running simulations with parameters drawn from a distribution. In practice, for any reproducible simulation project, users will write a script to generate the required command lines or input parameter files, invoke the simulation engine, and process the results in some way. This process is cumbersome and labor intensive, and a number of packages have been developed to allow simulations to be run directly in a high-level scripting language (Staab and Metzler 2016; Parobek *et al.* 2017; Gladstein *et al.* 2018).

The more recent trend has been to move away from this file and command-line driven approach and to instead provide direct interfaces to the simulation engines via an Application Programming Interface (API) (*e.g.*, Thornton 2014; Kelleher *et al.* 2016; Becheler *et al.* 2019; Haller and Messer 2019). The primary interface for msprime is through a thoroughly documented Python API, which has encouraged the development of an ecosystem of downstream tools (Terhorst *et al.* 2017; Chan *et al.* 2018; Spence and Song 2019; Adrion *et al.* 2020a, 2020b; Kamm *et al.* 2020; McKenzie and Eaton 2020; Montinaro *et al.* 2020; Rivera-Colón *et al.* 2021; Terasaki Hart *et al.* 2021). As well as providing a stable and efficient platform for building downstream applications, msprime’s Python API makes it much easier to build reproducible simulation pipelines, as the entire workflow can be encapsulated in a single script, and package and version dependencies explicitly stated using the pip or conda package managers. For example, the errors made in the influential simulation analysis of Martin *et al.* (2017) were only detected because the pipeline could be easily run and reanalyzed (Martin *et al.* 2020; Ragsdale *et al.* 2020).

**Figure 1:**
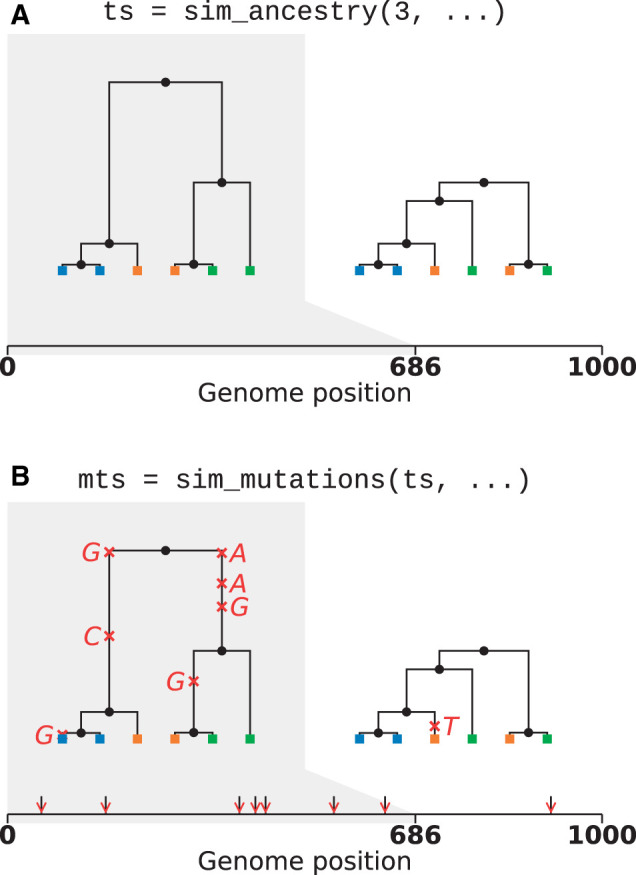
Visualization of the separation between ancestry and mutation simulation. (A) The result of an invocation of sim_ancestry is two trees along a 1 kb chunk of genome relating three diploid samples. Each diploid individual consists of two genomes (or nodes), indicated by color. (B) This ancestry is provided as the input to sim_mutations, which adds mutations. Graphics produced using tskit’s draw_svg method.

A major change for the msprime 1.0 release is the introduction of a new set of APIs, designed in part to avoid sources of error (see *Demography*) but also to provide more appropriate defaults while keeping compatibility with existing code. In the new APIs, ancestry and mutation simulation are fully separated (see Figure 1), with the sim_ancestry and sim_mutations functions replacing the legacy simulate function. Among other changes, the new APIs default to discrete genome coordinates and finite sites mutations, making the default settings more realistic and resolving a major source of confusion and error. The previous APIs are fully supported and tested, and will be maintained for the foreseeable future. The msp program (a command-line interface to the library) has been extended to include new commands for simulating ancestry and mutations separately. A particularly useful feature is the ability to specify demographic models in Demes format (Gower *et al.* 2022) from the command line, making simulation of complex demographies straightforward. We also provide an ms-compatible command-line interface to support existing workflows.

### Tree sequences

One of the key reasons for msprime’s substantial performance advantage over other simulators (Kelleher *et al.* 2016) is its use of the “succinct tree sequence” data structure to represent simulation results. The succinct tree sequence (usually abbreviated to “tree sequence”) was introduced by Kelleher *et al.* (2016) to concisely encode genetic ancestry and sequence variation and was originally implemented as part of msprime. We subsequently extracted the core tree sequence functionality from msprime to create the tskit library, which provides a large suite of tools for processing genetic ancestry and variation data via APIs in the Python and C languages (Tskit developers 2022). The availability of tskit as a liberally licensed (MIT) open source toolkit has enabled several other projects (*e.g.*, Haller and Messer 2019; Kelleher *et al.* 2019; Terasaki Hart *et al.* 2021; Wohns *et al.* 2021) to take advantage of the same efficient data structures used in msprime, and we hope that many more will follow. While a full discussion of tree sequences and the capabilities of tskit is beyond the scope of this article, we summarize some aspects that are important for simulation.

**Figure 2:**
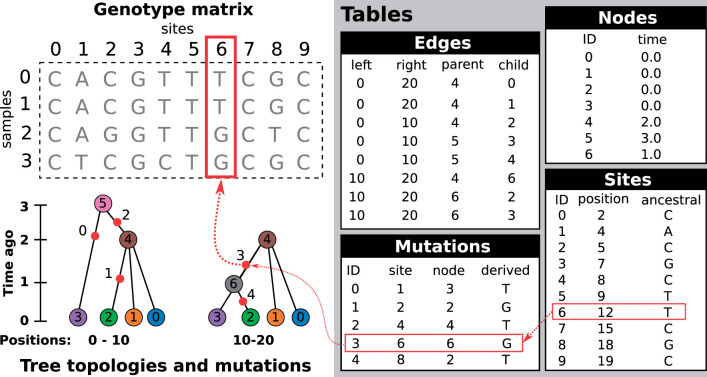
An example tree sequence describing genealogies and sequence variation for four samples at 10 sites on a chromosome of 20 bases long. Information is stored in a set of tables (the tables shown here include only essential columns, and much more information can be associated with the various entities). The node table stores information about sampled and ancestral genomes. The edge table describes how these genomes are related along a chromosome, and defines the genealogical tree at each position. The site and mutation tables together describe sequence variation among the samples. The genotype matrix and tree topologies shown on the left are derived from these tables.

Let us define a genome as the complete set of genetic material that a child inherits from one parent. Thus, a diploid individual has two (monoploid) genomes, one inherited from each parent. Since each diploid individual lies at the end of two distinct lineages of descent, they will be represented by *two* places (nodes) in any genealogical tree. In the tree sequence encoding a *node* therefore corresponds to a single genome, which is associated with its creation time (and other optional information), and recorded in a simple tabular format (Figure 2). Genetic inheritance between genomes (nodes) is defined by edges. An *edge* consists of a parent node, a child node, and the left and right coordinates of the contiguous chromosomal segment over which the child genome inherited genetic material from the parent genome. Parent and child nodes may correspond to ancestor and descendant genomes separated by many generations. Critically, edges can span multiple trees along the genome (usually referred to as “marginal” trees), and identical node IDs across different trees corresponds to the same ancestral genome. For example, in Figure 2, the branch from node 0 to 4 is present in both marginal trees, and represented by a single edge (the first row in the edge table). This simple device, of explicitly associating tree nodes with specific ancestral genomes and recording the contiguous segments over which parent–child relationships exist, generalizes the original “coalescence records” concept (Kelleher *et al.* 2016), and is the key to the efficiency of tree sequences (Kelleher *et al.* 2018, 2019; Ralph *et al.* 2020). Note that this formulation is fully compatible with the concept of an Ancestral Recombination Graph (ARG) and any ARG topology can be fully and efficiently encoded in the node and edge tables illustrated in Figure 2; see the *Ancestral Recombination Graphs* section below for more details.

The final output of most population genetic simulations is some representation of sequence variation among the specified samples. For coalescent simulations, we usually have three steps: (1) simulate the genetic ancestry, and optionally output the resulting marginal trees; (2) simulate sequence evolution conditioned on this ancestry by generating mutations (see the *Simulating mutations* section); and (3) output the resulting nucleotide sequences by percolating the effects of the mutations through the trees. Information about the mutations themselves—*e.g.*, where they have occurred on the trees—is usually not retained or made available for subsequent analysis. In msprime, however, we skip step (3), instead using tskit’s combined data model of ancestry and mutations to represent the simulated sequences. As illustrated in Figure 2, mutations are a fully integrated part of tskit’s tree sequence data model, and genetic variation is encoded by recording sites at which mutations have occurred, and where each mutation at those sites has occurred on the marginal tree. Crucially, the genome sequences themselves are never stored, or indeed directly represented in memory (although tskit can output the variant matrix in various formats, if required). It may at first seem inconvenient to have only this indirect representation of the genome sequences, but it is extremely powerful. First, the storage space required for simulations is dramatically reduced. For a simulation of *n* samples with *m* variant sites, we would require *O*(*nm*) space to store the sequence data as a variant matrix. However, if this simulation was of a recombining genome with *t* trees, then the tskit tree sequence encoding requires O(n+t+m) space, assuming we have *O*(1) mutations at each site (Kelleher *et al.* 2016). For large sample sizes, this difference is profound, making it conceivable, for example, to store the genetic ancestry and variation data for the entire human population on a laptop (Kelleher *et al.* 2019). As well as the huge difference in storage efficiency, it is often far more efficient to compute statistics of the sequence data from the trees and mutations than it is to work with the sequences themselves. For example, computing Tajima’s *D* from simulated data stored in the tskit format is several orders of magnitude faster than efficient variant matrix libraries for large sample sizes (Ralph *et al.* 2020).

The vast genomic datasets produced during the SARS-CoV-2 pandemic have highlighted the advantages of storing genetic variation data using the underlying trees. Turakhia *et al.* (2021) propose the Mutation Annotated Tree (MAT) format (consisting of a Newick tree and associated mutations in a binary format) and the matUtils program as an efficient way to store and process large viral datasets (McBroome *et al.* 2021), achieving excellent compression and processing performance. Similarly, phastsim (De Maio *et al.* 2021) was developed to simulate sequence evolution on such large SARS-CoV-2 phylogenies, and also outputs a Newick tree annotated with mutations (not in MAT format) to avoid the bottleneck of generating and storing the simulated sequences. While these methods illustrate the advantages of the general approach of storing ancestry and mutations rather than sequences, they do not generalize beyond their immediate settings, and no software library support is available.

The software ecosystem built around tskit is stable, mature, and rapidly growing. Simulators such as fwdpy11 (Thornton 2014), SLiM (Haller and Messer 2019), stdpopsim (Adrion *et al.* 2020a), Geonomics (Terasaki Hart *et al.* 2021), and GSpace (Virgoulay *et al.* 2021), and inference methods such as tsinfer (Kelleher *et al.* 2019), tsdate (Wohns *et al.* 2021), and Relate (Speidel *et al.* 2019) use either the Python or C APIs to support outputting results in tree sequence format. Tree sequences are stored in an efficient binary file format, and are fully portable across operating systems and processor architectures. The tskit library ensures interoperability between programs by having strict definitions of how the information in each of the tables is interpreted, and stringent checks for the internal consistency of the data model.

### Data analysis

The standard way of representing simulation data is to render the results in a text format, which must subsequently be parsed and processed as part of some analysis pipeline. For example, ms outputs a set of sequences and can also optionally output the marginal trees along the genome in Newick format, and variants of this approach are used by many simulators. Text files have many advantages, but are slow to process at scale. The ability to efficiently process simulation results is particularly important in simulation-based inference methods such as Approximate Bayesian Computation (ABC) (Beaumont *et al.* 2002; Csilléry *et al.* 2010; Wegmann *et al.* 2010) and machine learning-based approaches (Sheehan and Song 2016; Chan *et al.* 2018; Schrider and Kern 2018; Flagel *et al.* 2019; Sanchez *et al.* 2021). Clearly, simulation efficiency is crucial since the size and number of simulations that can be performed determines the depth to which one can sample from the model and parameter space. Equally important, however, is the efficiency with which the simulation results can be transformed into the specific input required by the inference method. In the case of ABC, this is usually a set of summary statistics of the sequence data, and methods avoid the bottleneck of parsing text-based file formats to compute these statistics by either developing their own simulators (*e.g*., Cornuet *et al.* 2008; Lopes *et al.* 2009) or creating forked versions (*i.e*., modified copies) of existing simulators (*e.g*., Thornton and Andolfatto 2006; Hickerson *et al.* 2007; Pavlidis *et al.* 2010; Huang *et al.* 2011; Quinto-Cortés *et al.* 2018), tightly integrated with the inference method. Modern approaches to ABC such as ABC-RF (Pudlo *et al.* 2016; Raynal *et al.* 2019) and ABC-NN (Blum and François 2010; Csilléry *et al.* 2012) use large numbers of weakly informative statistics, making the need to efficiently compute statistics from simulation results all the more acute. By using the stable APIs and efficient data interchange mechanisms provided by tskit, the results of an msprime simulation can be immediately processed, without format conversion overhead. The tskit library has a rich suite of population genetic statistics and other utilities, and is in many cases orders of magnitude faster than matrix-based methods for large sample sizes (Ralph *et al.* 2020). Thus, the combination of msprime and tskit substantially increases the overall efficiency of many simulation analysis pipelines.

Classical text-based output formats like ms are inefficient to process, but also lack a great deal of important information about the simulated process. The tree-by-tree topology information output by simulators in Newick format lacks any concept of node identity, and means that we cannot reliably infer information about ancestors from the output. Because Newick stores branch lengths rather than node times, numerical precision issues also arise for large trees (McGill *et al.* 2013). Numerous forks of simulators have been created to access information not provided in the output. For example, ms has been forked to output information about migrating segments (Rosenzweig *et al.* 2016), ancestral lineages (Chen and Chen 2013), and ms’s fork msHOT (Hellenthal and Stephens 2007) has in turn been forked to output information on local ancestry (Racimo *et al.* 2017). All of this information is either directly available by default in msprime, or can be optionally stored via options such as record_migrations or record_full_arg (see *Ancestral Recombination Graphs*) and can be efficiently and conveniently processed via tskit APIs.

### Simulating mutations

Because coalescent simulations are usually concerned with neutral evolution (see the *Selective sweeps* section*,* however), the problem of generating synthetic genetic variation can be decomposed into two independent steps: first, simulating genetic ancestry (the trees), then subsequently simulating variation by superimposing mutation processes on those trees (see Figure 1). A number of programs exist to place mutations on trees: for instance, the classical Seq-Gen program (Rambaut and Grassly 1997) supports a range of different models of sequence evolution, and various extensions to the basic models have been proposed (*e.g*., Cartwright 2005; Fletcher and Yang 2009). Partly for efficiency and partly in the interest of simplicity for users (*i.e*., to avoid intermediate text format conversions), population genetic simulators have tended to include their own implementations of mutation simulation, with most supporting the infinite sites model (*e.g*., Hudson 2002) but with several supporting a wide range of different models of sequence evolution (*e.g*., Mailund *et al.* 2005; Excoffier and Foll 2011; Virgoulay *et al.* 2021). Thus, despite the logical separation between the tasks of simulating ancestry and neutral sequence evolution, the two have been conflated in practice.

Part of the reason for this poor record of software reuse and modularity is the lack of standardized file formats, and in particular, the absence of common library infrastructure to abstract the details of interchanging simulation data. Although msprime also supports simulating both ancestry and mutations, the two aspects are functionally independent within the software; both ancestry and mutation simulators are present in msprime for reasons of convenience and history, and could be split into separate packages. The efficient C and Python interfaces for tskit make it straightforward to add further information to an existing file, and because of its efficient data interchange mechanisms, there is no performance penalty for operations being performed in a different software package. Thanks to this interoperability, msprime’s mutation generator can work with *any*tskit tree sequence, be it simulated using SLiM (Haller and Messer 2019) or fwdpy11 (Thornton 2014), or estimated from real data (Kelleher *et al.* 2019; Speidel *et al.* 2019; Wohns *et al.* 2021). It is a modular component intended to fit into a larger software ecosystem, and is in no way dependent on msprime’s ancestry simulator.

We have greatly extended the sophistication of msprime’s mutation generation engine for version 1.0, achieving near feature-parity with Seq-Gen. We support a large number of mutation models, including the JC69 (Jukes and Cantor 1969), F84 (Felsenstein and Churchill 1996), and GTR (Tavaré 1986) nucleotide models and the BLOSUM62 (Henikoff and Henikoff 1992) and PAM (Dayhoff *et al.* 1978) amino acid models. Other models, such as the Kimura two and three parameter models (Kimura 1980, 1981), can be defined easily and efficiently in user code by specifying a transition matrix between any number of alleles. Mutation rates can vary along the genome, and multiple mutation models can be imposed on a tree sequence by overlaying mutations in multiple passes. We have extensively validated the results of mutation simulations against both theoretical expectations and output from Seq-Gen (Rambaut and Grassly 1997) and Pyvolve (Spielman and Wilke 2015).

**Figure 3:**
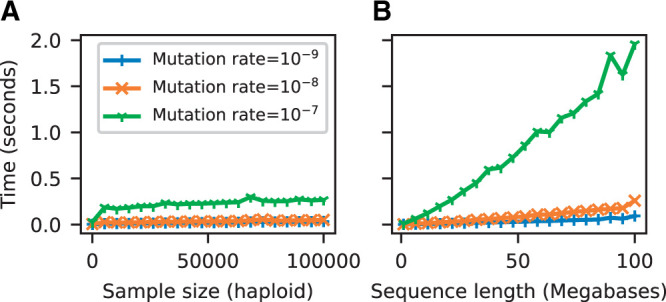
Time required to run sim_mutations on tree sequences generated by sim_ancestry (with a population size of 10^4^ and recombination rate of $10^{-8}$) for varying (haploid) sample size and sequence length. We ran 10 replicate mutation simulations each for three different mutation rates, and report the average CPU time required (Intel Core i7-9700). (A) Holding sequence length fixed at 10 megabases and varying the number of samples (tree tips) from 10 to 100,000. (B) Holding number of samples fixed at 1000, and varying the sequence length from 1 to 100 megabases.

Simulating mutations in msprime is efficient. Figure 3 shows the time required to generate mutations (using the default JC69 model) on simulated tree sequences for a variety of mutation rates as we vary the number of samples (Figure 3A) and the sequence length (Figure 3B). For example, the longest running simulation in Figure 3B required less than 2 s to generate an average of 1.5 million mutations over 137,081 trees in a tree sequence with 508,125 edges. This efficiency for large numbers of trees is possible because the tree sequence encoding allows us to generate mutations on an edge-by-edge basis (see Figure 2 and the Mutation generation section in Appendix), rather than tree-by-tree and branch-by-branch as would otherwise be required. Simulating mutations on a single tree is also very efficient; for example, we simulated mutations under the BLOSUM62 amino acid model for a tree with 10^6^ leaves over 10^4^ sites (resulting in ∼260,000 mutations) in about 0.8 s, including the time required for file input and output. We do not attempt a systematic benchmarking of msprime’s mutation generation code against other methods, because at this scale, it is difficult to disentangle the effects of inefficient input and output formats from the mutation generation algorithms. Given the above timings, it seems unlikely that generating mutations with msprime would be a bottleneck in any realistic analysis.

There are many ways in which the mutation generation code in msprime could be extended. For example, we intend to add support for microsatellites (Mailund *et al.* 2005), codon models (Arenas and Posada 2007), and indels (Cartwright 2005; Fletcher and Yang 2009), although changes may be required to tskit’s data model which is currently based on the assumption of independent sites.

### Recombination

Crossover recombination is implemented in msprime using Hudson’s algorithm, which works backwards in time, generating common ancestor and recombination events and tracking their effects on segments of ancestral material inherited from the sample (Hudson 1983a, 1990; Kelleher *et al.* 2016). Common ancestor events merge the ancestral material of two lineages, and result in coalescences in the marginal trees when ancestral segments overlap. Recombination events split the ancestral material for some lineage at a breakpoint, creating two independent lineages. Using the appropriate data structures (Kelleher *et al.* 2016), this process is much more efficient to simulate than the equivalent left-to-right approach (Wiuf and Hein 1999a, 1999b). In msprime 1.0, recombination rates can vary along a chromosome, allowing us to simulate recombination hotspots and patterns of recombination from empirical maps. The implementation of recombination in msprime is extensively validated against analytical results (Hudson 1983a; Kaplan and Hudson 1985) and simulations by ms, msHOT, and SLiM.

The Sequentially Markovian Coalescent (SMC) is an approximation of the coalescent with recombination (McVean and Cardin 2005; Marjoram and Wall 2006), and was primarily motivated by the need to simulate longer genomes than was possible using tools like ms. The SMC is a good approximation to the coalescent with recombination when we have fewer than five sampled genomes (Hobolth and Jensen 2014; Wilton *et al.* 2015), but the effects of the approximation are less well understood for larger sample sizes, and several approaches have been proposed that allow simulations to more closely approximate the coalescent with recombination (Chen *et al.* 2009; Wang *et al.* 2014; Staab *et al.* 2015). The SMC and SMC’ models are supported in msprime 1.0. However, they are currently implemented using a naive rejection sampling approach, and are somewhat slower to simulate than the exact coalescent with recombination. These models are therefore currently only appropriate for studying the SMC approximations themselves, although we intend to implement them more efficiently in future versions.

In human-like parameter regimes and for large sample sizes, msprime’s implementation of the exact coalescent with recombination comprehensively outperforms all other simulators, including those based on SMC approximations (Kelleher *et al.* 2016). However, it is important to note that although the implementation of Hudson’s algorithm is very efficient, it is still quadratic in the population scaled recombination rate $\rho =4N_{e}L$, where *L* is the length of the genome in units of recombination distance. This is because Hudson’s algorithm tracks recombinations not only in segments ancestral to the sample, but also between ancestral segments. As mentioned above, common ancestor events in which the ancestral material of two lineages is merged only result in coalescences in the marginal trees if their ancestral segments overlap. If there is no overlap, the merged segments represent an ancestral chromosome that is a genetic ancestor of the two lineages, but not the most recent common genetic ancestor at any location along the genome. When this happens, the merged lineage carries “trapped” genetic material that is not ancestral to any samples, but where recombinations can still occur (Wiuf and Hein 1999b). For large *ρ*, recombination events in trapped ancestral material will dominate, and so we can use this as a proxy for the overall number of events in Hudson’s algorithm. Hein *et al.* (2004, Equation 5.10) gave

(1) $$\rho (\rho +1){\left(\sum_{i=1}^{n-1}\frac{1}{i}\right)}^{2}$$

as an upper bound on the number of recombination events within trapped ancestral material for *n* samples. As discussed in the Time complexity of Hudson’s algorithm Appendix, the quadratic dependence of simulation running time on *ρ* implied by Equation (1) is well supported by observations, and provides a useful means of predicting how long a particular simulation might require.

### Gene conversion

Gene conversion is a form of recombination that results in the transfer of a short segment of genetic material, for example between homologous chromosomes (Chen *et al.* 2007). Since gene conversion impacts much shorter segments than crossover recombination (typically below 1 kb), it affects patterns of linkage disequilibrium differently (Korunes and Noor 2017). Wiuf and Hein (2000) modeled gene conversion in the coalescent via a rate at which gene conversion events are initiated along the genome and a geometrically distributed tract length. In terms of the ancestral process, gene conversion differs from crossover recombination (as described in the previous section) in that it extracts a short tract of ancestry into an independent lineage, rather than splitting ancestry to the left and right of a given breakpoint. We have implemented this model of gene conversion in msprime 1.0, and validated the output against ms and analytical results (Wiuf and Hein 2000).

**Figure 4:**
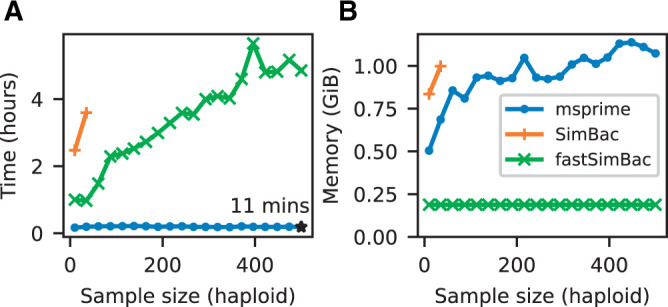
Comparison of simulation performance using msprime (sim_ancestry), SimBac, and fastSimBac for varying (haploid) sample sizes, and the current estimates for *E. coli* parameters (Lapierre *et al.* 2016): a 4.6 Mb genome, $N_{e}=1.8\times 10^{8}$, gene conversion rate of $8.9\times 10^{-11}$ per base and mean tract length of 542. We report (A) the total CPU time and (B) maximum memory usage averaged over five replicates (Intel Xeon E5-2680 CPU). We did not run SimBac beyond first two data points because of the very long running times.

Gene conversion is particularly useful to model homologous recombination in bacterial evolution, and so we compare the performance of msprime with gene conversion to two specialized bacterial simulators, SimBac (Brown *et al.* 2016) and fastSimBac (De Maio and Wilson 2017). Figure 4A shows that msprime is far more efficient than both SimBac and the SMC-based approximation fastSimBac. Figure 4B shows that msprime requires somewhat more memory than fastSimBac (as expected since fastSimBac uses a left-to-right SMC approximation), but is still reasonably modest at around 1 GiB for a simulation of 500 whole *Escherichia* *coli* genomes. However, msprime is currently lacking many of the specialized features required to model bacteria, and so an important avenue for future work is to add features such as circular genomes and bacterial gene transfer (Baumdicker and Pfaffelhuber 2014).

### Demography

One of the key applications of population genetic simulations is to generate data for complex demographies. Beyond idealized cases such as stepping-stone or island models, or specialized cases such as isolation-with-migration models, analytical results are rarely possible. Simulation is therefore integral to the development and evaluation of methods for demographic inference. The demography model in msprime is directly derived from the approach used in ms, and supports an arbitrary number of randomly mating populations exchanging migrants at specified rates. A range of demographic events are supported, which allow for varying population sizes and growth rates, changing migration rates over time, as well as population splits, admixtures, and pulse migrations.

A major change for msprime 1.0 is the introduction of the new Demography API, designed to address a design flaw in the msprime 0.x interface which led to avoidable errors in downstream simulations (Ragsdale *et al.* 2020). The new API is more user-friendly, providing the ability, for example, to refer to populations by name rather than their integer identifiers. We also provide numerical methods to compute the coalescence rates for two or more lineages which can be inverted to obtain the “inverse instantaneous coalescence rate” of Chikhi *et al.* (2018). Many popular approaches in population genetics use the distribution of coalescence rates between pairs of lineages to infer effective population sizes over time (Li and Durbin 2011; Sheehan *et al.* 2013; Schiffels and Durbin 2014) or split times and subsequent migration rates between populations (Wang *et al.* 2020). These numerical methods provide a valuable ground-truth when evaluating such inference methods, as illustrated by Adrion *et al.* (2020a).

### Instantaneous bottlenecks

A common approach to modeling the effect of demographic history on genealogies is to assume that effective population size (*N_e_*) changes in discrete steps which define a series of epochs (Griffiths *et al.* 1994; Marth *et al.* 2004; Keightley and Eyre-Walker 2007; Li and Durbin 2011). In this setting of piecewise constant *N_e_*, capturing a population bottleneck requires three epochs: *N_e_* is reduced by some fraction *b* at the start of the bottleneck, *T_start_*, and recovers to its initial value at time *T_end_* (Marth *et al.* 2004). If bottlenecks are short both on the timescale of coalescence and mutations, there may be little information about the duration of a bottleneck ($T_{\mathrm{end}}-T_{\mathrm{start}}$) in sequence data. Thus a simpler, alternative model is to assume that bottlenecks are instantaneous ($T_{\mathrm{end}}-T_{\mathrm{start}}\to 0$) and generate a sudden burst of coalescence events (a multiple merger event) in the genealogy. The strength of the bottleneck *B* can be thought of as an (imaginary) time period during which coalescence events are collapsed, *i.e*., there is no growth in genealogical branches during *B* and the probability that a single pair of lineages entering the bottleneck coalesce during the bottleneck is $1-e^{-B}$. Although this simple two-parameter model of bottlenecks is attractive and both analytic results and empirical inference methods (Griffiths *et al.* 1994; Galtier *et al.* 2000; Birkner *et al.* 2009; Bunnefeld *et al.* 2015) have been developed under this model, there has been no software available to simulate data under instantaneous bottleneck histories.

We have implemented instantaneous bottlenecks in msprime 1.0 using a variant of Hudson’s linear time single-locus coalescent algorithm (Hudson 1990), and validated the results by comparing against analytical expectations (Bunnefeld *et al.* 2015).

### Multiple merger coalescents

Kingman’s coalescent assumes that only two ancestral lineages can merge at each merger event. Although this is generally a reasonable approximation, there are certain situations in which the underlying mathematical assumptions are violated; for example, in certain highly fecund organisms (Beckenbach 1994; Hedgecock 1994; Árnason 2004; Hedgecock and Pudovkin 2011; Irwin *et al.* 2016; Vendrami *et al.* 2021), where individuals have the ability to produce numbers of offspring on the order of the population size and therefore a few individuals may produce the bulk of the offspring in any given generation (Hedgecock 1994). These population dynamics violate basic assumptions of the Kingman coalescent, and are better modeled by “multiple-merger” coalescents (Donnelly and Kurtz 1999; Pitman 1999; Sagitov 1999; Schweinsberg 2000; Möhle and Sagitov 2001), in which more than two lineages can merge in a given event. Multiple-merger coalescent processes have also been shown to be relevant for modeling the effects of selection on gene genealogies (Gillespie 2000; Durrett and Schweinsberg 2004; Desai *et al.* 2013; Neher and Hallatschek 2013; Schweinsberg 2017).

Although multiple merger coalescents have been of significant theoretical interest for around two decades, there has been little practical software available to simulate these models. Kelleher *et al.* (2013, 2014) developed packages to simulate a related spatial continuum model (Barton *et al.* 2010), Zhu *et al.* (2015) simulate genealogies within a species tree based on a multiple-merger model, and Becheler and Knowles (2020) provide a general method for simulating multiple merger processes as part of the Quetzal framework (Becheler *et al.* 2019). The Beta-Xi-Sim simulator (Koskela 2018; Koskela and Berenguer 2019) also includes a number of extensions to the Beta-coalescent. None of these methods work with large genomes, and very little work has been performed on simulating multiple merger processes with recombination.

We have added two multiple merger coalescent models in msprime 1.0, the Beta-coalescent (Schweinsberg 2003), and “Dirac”-coalescent (Birkner *et al.* 2013a), allowing us to efficiently simulate such models with recombination for the first time. These simulation models have been extensively validated against analytical results from the site frequency spectrum (Birkner *et al.* 2013b; Blath *et al.* 2016; Hobolth *et al.* 2019) as well as more general properties of coalescent processes. See the Multiple merger coalescent model in Appendix for more details and model derivations.

### Ancestral Recombination Graphs

The ARG was introduced by Griffiths (Griffiths 1991; Griffiths and Marjoram 1997) to represent the stochastic process of the coalescent with recombination as a graph. This formulation is complementary to Hudson’s earlier work (Hudson 1983a), and substantially increased our theoretical understanding of recombination. In Griffiths’ ARG formulation, a realization of the coalescent with recombination is a graph in which vertices represent common ancestor or recombination events, and edges represent lineages. There is the “big” ARG, in which we track lineages arising out of recombinations regardless of whether they carry ancestral material (Ethier and Griffiths 1990), and the “little” ARG in which we only track genetic ancestors. Over time, usage of the term has shifted away from its original definition as a stochastic process, to being interpreted as a representation of a particular genetic ancestry as a graph, without necessarily following the specific details of the Griffiths formulation (*e.g*., Minichiello and Durbin 2006; Mathieson and Scally 2020). Under the latter interpretation, the tree sequence encoding of genetic ancestry (described above) clearly *is* an ARG: the nodes and edges define a graph in which edges are annotated with the set of disjoint genomic intervals through which ancestry flows.

For our purposes, an ARG is a realization of the coalescent with recombination, in the Griffiths (little ARG) sense. As described in detail by Kelleher *et al.* (2016), Hudson’s algorithm works by dynamically traversing a little ARG. The graph is not explicitly represented in memory, but is partially present through the extant lineages and the ancestral material they carry over time. We do not output the graph directly, but rather store the information required to recover the genealogical history as nodes and edges in a tree sequence. This is far more efficient than outputting the simulated ARG in its entirety. For a given scaled recombination rate *ρ* (setting aside the dependency on the sample size *n*), we know from Equation (1) that the number of nodes in an ARG is $O(\rho^{2})$, whereas the size of the tree sequence encoding is O(ρ) (Kelleher *et al.* 2016). This difference between a quadratic and a linear dependency on *ρ* is profound, and shows why large simulations cannot output an ARG in practice.

**Figure 5:**
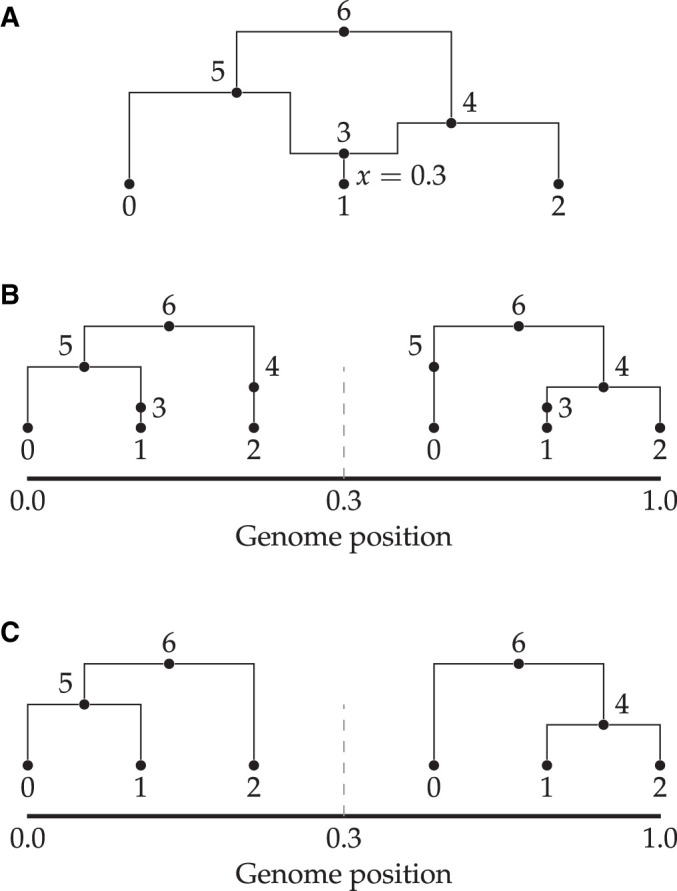
(A) A simple ARG in which a recombination occurs at position 0.3; (B) the equivalent topology depicted as a tree sequence, including the recombination node; (C) the same tree sequence topology “simplified” down to its minimal tree sequence representation. Note the original node IDs have been retained for clarity.

Although by default msprime outputs tree sequences that contain full information about the genealogical trees, their correlation structure along the chromosome, and the ancestral genomes on which coalescences occurred, some information is lost in this mapping down from ARG space to the minimal tree sequence form. In particular, we lose information about ancestral genomes that were common ancestors but in which no coalescences occurred, and also information about the precise time and chromosomal location of recombination events. In most cases, such information is of little relevance as it is in principle unknowable, but there are occasions such as visualization or computing likelihoods in which it is useful. We therefore provide the record_full_arg option in msprime to store a representation of the complete ARG traversed during simulation. This is done by storing extra nodes (marked with specific flags, so they can be easily identified later) and edges in the tree sequence (Figure 5). One situation in which a record of the full ARG is necessary is when we wish to compute likelihoods during inference. The likelihood is a central quantity in evaluating the plausibility of a putative ancestry as an explanation of DNA sequence data, both directly through *e.g*., approaches based on maximum likelihood, and as an ingredient of methods such as Metropolis–Hastings (Kuhner *et al.* 2000; Nielsen 2000; Wang and Rannala 2008). We provide functions to compute the likelihood of ARG realizations and mutational patterns under the standard coalescent and infinite sites mutation model. For details, see the Appendix: Likelihood calculations.

### Selective sweeps

Another elaboration of the standard neutral coalescent with recombination is the addition of selective sweeps (Kaplan *et al.* 1989; Braverman *et al.* 1995; Kim and Stephan 2002). Sweeps are modeled by creating a structured population during the sojourn of the beneficial mutation through the population (*i.e*., the sweep phase) in which lineages may transit between favored and unfavored backgrounds through recombination. This approach allows for many selective sweep scenarios to be simulated efficiently, including recurrent, partial, and soft selective sweeps. However, this efficiency comes at the cost of flexibility in comparison to forwards in time simulation. Several specialized simulators have been developed to simulate sweeps in the coalescent, including SelSim (Spencer and Coop 2004), mbs (Teshima and Innan 2009), msms (Ewing and Hermisson 2010), cosi2 (Shlyakhter *et al.* 2014), and discoal (Kern and Schrider 2016).

Selective sweeps are implemented in the coalescent as a two-step process: first generating an allele frequency trajectory, and then simulating a structured coalescent process conditioned on that trajectory. Following discoal, we generate sweep trajectories in msprime using a jump process approximation to the conditional diffusion of an allele bound for fixation (Coop and Griffiths 2004), as detailed in the Selective sweeps model in Appendix. Given a randomly generated allele frequency trajectory, the simulation of a sweep works by assigning lineages to two different structured coalescent “labels,” based on whether they carry the beneficial allele. The allele frequency trajectory determines the relative sizes of the “populations” in these labels over time, and therefore the rates at which various events occur. Common ancestor events can then only merge lineages from *within* a label, but lineages can transfer from one label to the other (*i.e*., from the advantageous to disadvantageous backgrounds, and vice versa) as a result of recombination events. Once we have reached the end of the simulated trajectory, the sweep is complete, and we remove the structured coalescent labels. Simulation may then resume under any other ancestry model.

**Figure 6:**
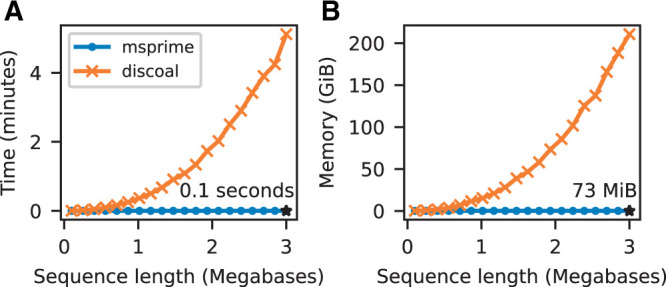
Comparison of selective sweep simulation performance in msprime (sim_ancestry) and discoal (Intel Xeon Gold 6148 CPU). We report the average CPU time and maximum memory usage when simulating three replicates for 100 diploid samples in a model with a single selective sweep in its history, where the beneficial allele had a selection coefficient of *s *=* *0.05, a per-base recombination rate of $10^{-8}$, population size of $N=10^{4}$, and sequence length varying from 100 kb–3000 kb.

Figure 6 compares the performance of msprime and discoal under a simple sweep model, and shows that msprime has far better CPU time and memory performance. Since our implementation uses the abstract label system mentioned above, adding support for similar situations, such as inversions (Peischl *et al.* 2013), should be straightforward.

### Discrete time Wright–Fisher

The coalescent is an idealized model and makes many simplifying assumptions, but it is often surprisingly robust to violations of these assumptions (Wakeley *et al.* 2012). One situation in which the model does break down is the combination of large sample size and long recombining genomes, where the large number of recombination events in the recent past results in more than the biologically possible $2^{t}$ ancestors in *t* diploid generations (Nelson *et al.* 2020). This pathological behavior results in identity-by-descent, long-range linkage disequilibrium, and ancestry patterns deviating from Wright–Fisher expectations, and the bias grows with larger sample sizes (Wakeley *et al.* 2012; Bhaskar *et al.* 2014; Nelson *et al.* 2020). Precisely this problem occurs when simulating modern human datasets, and we have implemented a Discrete Time Wright–Fisher (DTWF) model in msprime to address the issue. The DTWF simulates backwards in time generation-by-generation so that each gamete has a unique diploid parent, and multiple recombinations within a generation results in crossover events between the same two parental haploid copies. The method is described in detail by Nelson *et al.* (2020).

**Figure 7:**
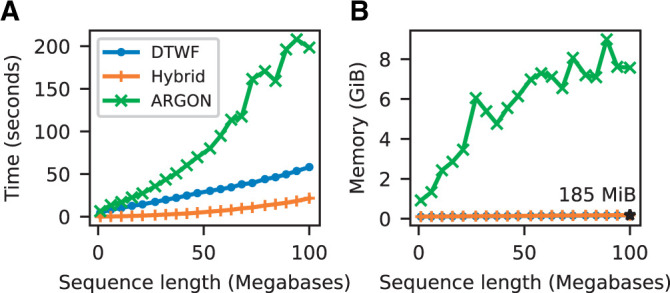
Comparison of DTWF simulation performance in msprime (sim_ancestry) and ARGON (Intel Xeon E5-2680 CPU). We ran simulations with a population size of 10^4^ and recombination rate of $10^{-8}$, with 500 diploid samples and varying sequence length. We report (A) total CPU time and (B) maximum memory usage; each point is the average over five replicate simulations. We show observations for ARGON, msprime’s DTWF implementation (“DTWF”) and a hybrid simulation of 100 generations of the DTWF followed by the standard coalescent with recombination (“Hybrid”). We ran ARGON with a mutation rate of 0 and with minimum output options, with a goal of measuring only ancestry simulation time. Memory usage for msprime’s DTWF and hybrid simulations are very similar.

Figure 7 shows that msprime simulates the DTWF more quickly and requires substantially less memory than ARGON (Palamara 2016), a specialized DTWF simulator. However, the generation-by-generation approach of the DTWF is less efficient than the coalescent with recombination when the number of lineages is significantly less than the population size (the regime where the coalescent is an accurate approximation), which usually happens in the quite recent past (Bhaskar *et al.* 2014). We therefore support changing the simulation model during a simulation so that we can run hybrid simulations, as proposed by Bhaskar *et al.* (2014). Any number of different simulation models can be combined, allowing for the flexible choice of simulation scenarios. As the DTWF improves accuracy of genealogical patterns in the recent past, we can simulate the recent history using this model and then switch to the standard coalescent to more efficiently simulate the more ancient history.

### Integration with forward simulators

A unique feature of msprime is its ability to simulate genetic ancestries by extending an existing partial genetic ancestry. Given a tree sequence that is complete up until time *t* ago as input (where marginal trees may or may not have fully coalesced), msprime can efficiently obtain the segments of ancestral material present at this time, and then run the simulation backwards in time from there. This allows a simulated ancestry to be produced by any number of different processes across disjoint time slices. In practice, this feature is used to “complete” forwards-time ancestry simulations (Kelleher *et al.* 2018) that may have not fully coalesced. This process (“recapitation”) can be orders of magnitude faster than the standard approach of neutral burn-in; see Haller *et al.* (2018) for more details and examples. This interoperability between simulators, where a partial ancestry simulation produced by SLiM (Haller and Messer 2019) or fwdpy11 (Thornton 2014) can be picked up and completed by another simulator, with complete information retained—at scale—is unprecedented. There may be an opportunity for other forward genetic simulators (*e.g*., Gaynor *et al.* 2021) to leverage the tree sequence data format and associated tools.

### Development model

Msprime has a large number of features, encompassing the functionality of several more specialized simulators while maintaining excellent performance. It is developed by a geographically distributed team of volunteers under an open source community development model, with a strong emphasis on code quality, correctness, good documentation, and inclusive development. As in any large code base, unit tests play a key role in ensuring that new additions behave as expected and msprime has an extensive suite. These tests are run automatically on different operating systems on each pull request (where a contributor proposes a code change), using standard Continuous Integration (CI) methodology. Other CI services check for common errors, code formatting issues, and produce reports on the level of test coverage for the proposed change.

Unit tests are vital for ensuring software quality and correctness, but they are usually of little value in assessing the statistical properties of simulations. To validate the correctness of simulation output, we maintain a suite of statistical tests (as of 1.0.0, 217 validation tests). These consist of running many replicate simulations to check the properties of the output against other simulators, and where possible against analytical results. For example, simulations of complex demography are validated against ms, selective sweeps against discoal, and Wright–Fisher simulations against forwards in time simulations in SLiM. This suite of tests is run before every release, to ensure that statistical errors have not been introduced.

More visibly to the end user, we also have a high standard for documentation, with precise, comprehensive, and cross-linked documentation that is automatically built from the code base and served through the website https://tskit.dev. With the goal of lowering the entry barrier to new users, we have invested significant effort in writing examples and introductions, and making common tasks discoverable. We also view contributions to documentation as equally important to the project as writing code or designing methods: what use would it be to write reliable, stable software if no-one used it?

An important goal of msprime’s development model is to maximize accessibility for prospective users and contributors, and to encourage diversity in our community. Gender and racial inequality caused by discrimination and marginalization is a major problem across the sciences (Wellenreuther and Otto 2016; Shannon *et al.* 2019) and in open source software development (Trinkenreich *et al.* 2021). Within our field, the contribution of women to early computational methods in population genetics was marginalized (Dung *et al.* 2019), and women continue to be under-represented in computational biology (Bonham and Stefan 2017). The authorship of our paper reflects these trends, with a skew toward men and affiliations in the United States and Europe. We know the importance of creating and strengthening networks to develop and maintain a diverse community of contributors, and we are committed to fostering a supportive and collaborative environment that helps to address these inequalities in our field.

## Discussion

The 1.0 release of msprime marks a major increase in the breadth of available features and the potential biological realism of simulations. These abilities will allow researchers to perform more robust power analyses, more reliably test new methods, carry out more reliable inferences, and more thoroughly explore the properties of theoretical models. Despite this complexity and generality, msprime’s performance is state-of-the-art and all features are extensively tested and statistically validated. These advances have only been possible thanks to a distributed, collaborative model of software development, and the work of many people.

Even though simulation has long been a vital tool in population genetics, such collaborative software development has historically been uncommon. A huge proliferation of tools have been published (the references here are not exhaustive) and only a small minority of these are actively developed and maintained today. The ecosystem is highly fragmented, with numerous different ways of specifying parameters and representing results, and there are significant software quality issues at all stages. This is unsurprising, since the majority of simulation software development is performed by students, often without formal training in software development. The result resembles Haldane’s sieve for new mutations: many new pieces of software stay permanently on a dusty shelf of supplementary materials, while some of those that prove particularly useful when new (like dominant alleles) are quickly adopted. Although this has produced many good tools and enabled decades of research, it also represents a missed opportunity to invest as a community in shared infrastructure and mentorship in good software development practice.

Scientific software is vital apparatus, and must be engineered to a high quality if we are to trust its results. There is a growing realization across the sciences (*e.g*., Siepel 2019; Harris *et al.* 2020; Gardner *et al.* 2021) that investing in shared community infrastructure produces better results than a proliferation of individually maintained tools, allowing scientists to focus on their specific questions rather than software engineering. Msprime 1.0 is the result of such a community process, with features added by motivated users, taking advantage of the established development practices and infrastructure. Software development in a welcoming community, with mentorship by experienced developers, is a useful experience for many users. The skills that contributors learn can lead to greatly increased productivity in subsequent work (*e.g*., through more reliable code and better debugging skills). We hope that users who find that features they require are missing will continue to contribute to msprime, leading to a community project that is both high quality and sustainable in the long term.

The succinct tree sequence data structure developed for msprime provides a view of not only genetic variation, but also the genetic ancestry that produced that variation. Recent breakthroughs in methods to infer genetic ancestry in recombining organisms (Rasmussen *et al.* 2014; Kelleher *et al.* 2019; Speidel *et al.* 2019, 2021; Schaefer *et al.* 2021; Wohns *et al.* 2021) have made it possible to estimate such ancestry from real data at scale for the first time (Harris 2019; Tang 2019). Given such inferred ancestry, many exciting applications become possible. For example, Osmond and Coop (2021) developed a method to estimate the location of genetic ancestors based on inferred trees, and other uses are sure to follow. Since the inferred genetic ancestry becomes the input for other downstream inferences, it is vitally important that these primary inferences are thoroughly validated, with the detailed properties of the inferred ancestries cataloged and understood. Msprime will continue to be an important tool for these inferences and validations, and in this context the ability to interoperate with other methods—particularly forwards simulators—through the succinct tree sequence data structure and tskit library will be essential.

## Data availability

Msprime is freely available under the terms of the GNU General Public License v3.0, and can be installed from the Python Package Index or the conda-forge (conda-forge community 2015) conda channel. Development is conducted openly on GitHub at https://github.com/tskit-dev/msprime/. The documentation for msprime is available at https://tskit.dev/msprime/docs/. The source code for all the evaluations and figures in this manuscript is available at https://github.com/tskit-dev/msprime-1.0-paper/.

## Figures and Tables

**Figure 8:**
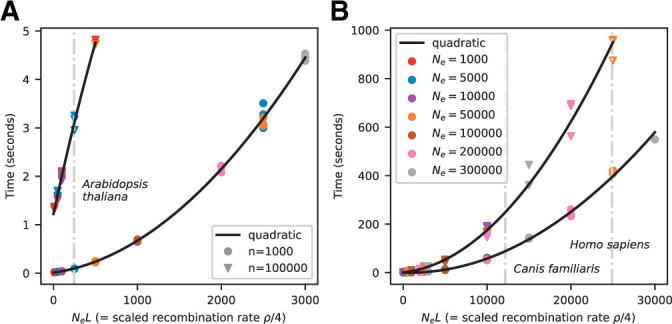
Running time of sim_ancestry for (A) small and (B) larger simulations on an Intel i7-6600U CPU. Each point is the run time of one simulation, for various values of effective population size (*N_e_*), chromosome length in Morgans (*L*), and number of diploid samples (*n*). Run time scales quadratically with the product of *N_e_* and *L*, shown on the horizontal axis. For example, (A) shows that 1000 samples of 1 Morgan-length chromosomes from a population of $N_{e}=2,000$ diploids would take about 2 s, and (equivalently) that the same number of 0.01 Morgan segments with $N_{e}=200,000$ would take the same time. Since recombination rate in these simulations was $10^{-8}$, *L* is the number of base pairs divided by 10^8^. The black lines are quadratic fits separately in each panel and sample size. Vertical gray lines show the approximate values of $N_{e}L$ for chromosome 1 in three species, using values from the stdpopsim catalog (Adrion *et al.* 2020a).

## Appendix

### Mutation generation

The algorithm that msprime uses to simulate mutations on a tree sequence proceeds in two steps: first, mutations are “placed” on the tree sequence (*i.e*., sampling their locations in time, along the genome, and on the marginal tree), and then the ancestral and derived alleles of each mutation are generated. All mutation models share the code to place mutations, but choose alleles in different ways.

First, mutations are placed on the tree sequence under an inhomogeneous Poisson model by applying them independently to each edge. If an edge spans a region [a,b) of the genome and connected parent and child nodes with times *s *<* t*, and the mutation rate locally is *μ*, then the number of mutations on the edge is Poisson with mean μ(t−s)(b−a), and each mutation is placed independently at a position chosen uniformly in [a,b) and a time uniformly in [s,t). In a discrete genome, all positions are integers and so more than one mutation may occur at the same position on the same edge. Otherwise (*i.e*., for an infinite-sites model), positions are rejection sampled to obtain a unique floating-point number. If an edge spans a region of the genome with more than one mutation rate, this is done separately for each sub-region on which the mutation rate is constant. Since each edge is processed independently, the algorithm scales linearly with the number of edges in the tree sequence.

Next, alleles are chosen for each mutation. If the site was not previously mutated, then a new ancestral allele is chosen for the site, according to an input distribution of ancestral state allele probabilities. Then, each mutation on the tree is considered in turn, and a derived allele is randomly chosen based on the parental allele (which may be the ancestral allele or the derived allele of a previous mutation). Finally, information about the mutations are recorded in the site and mutation tables of the tree sequence.

A mutation model must, therefore, provide two things: a way of choosing an ancestral allele for each new variant site, and a way of choosing a derived allele given the parental allele at each mutation. Perhaps the simplest mutation model implemented in msprime is the InfiniteAlleles mutation model, which keeps an internal counter so that the requested alleles are assigned subsequent (and therefore unique) integers.

The distribution of ancestral alleles is used to choose the allele present at the root of the tree at each mutated site, *i.e*., the root_distribution. Mutation models with a finite possible set of alleles have a natural choice for this distribution—the *stationary distribution* of the mutation process. (All mutation models are Markovian, so this may be found as the top left eigenvector of the mutation matrix.) This is the default in most models, except, *e.g.*, the BinaryMutationModel, whose alleles are 0 and 1 and always labels the ancestral allele “0.” However, mutational processes are not in general stationary, so we often allow a different root distribution to be specified.

Since the general algorithm above applies mutations at a single rate independent of ancestral state, a model in which different alleles mutate at different rates must necessarily produce some silent mutations, *i.e*., mutations in which the derived allele is equal to the parental allele. To illustrate this, consider a mutation model in which *A* or *T* mutates to a randomly chosen different nucleotide at rate *α* and *C* or *G* mutates at rate *β*, with β<α. To implement this, first place mutations at the largest total rate, which is *α*. Then, at each site, choose an ancestral allele from the root distribution, and for each mutation, choose a derived allele as follows: if the parental allele is *A* or *T*, then choose a random derived allele different to the parental allele; if the parental allele is *C* or *G*, then choose the derived allele to be equal to the parent allele with probability 1-β/α, and randomly choose a different nucleotide otherwise. This produces the correct distribution by Poisson thinning: a Poisson process with rate *α* in which each point is kept independently with probability β/α is equivalent to a Poisson process with rate *β*. All finite-state models (implemented under the generic MatrixMutationModel class) work in this way: mutations are placed at the maximum mutation rate, and then some silent mutations will result.

In previous versions of msprime, silent mutations were disallowed, and we could have removed them from the output entirely. However, we have chosen to leave them in, so that for instance simulating with the HKY mutation model will result in silent mutations if not all equilibrium frequencies are the same. The presence of silent mutations may at first be surprising but there is a good reason to leave them in: to allow layering of different mutation models. Suppose that we wanted to model the mutation process as a mixture of more than one model, *e.g*., Jukes–Cantor mutations at rate *μ*_1_, and HKY mutations occur at rate *μ*_2_. Layering multiple calls to sim_mutations is allowed, so we could first apply mutations with the JC69 model at rate *μ*_1_ and then add more with the HKY model at rate *μ*_2_. However, there is a small statistical problem: suppose that after applying Jukes–Cantor mutations, we have an A→C mutation, but then the HKY mutations inserts another mutation in the middle, resulting in A→C→C. If neither mutation model allows silent transitions, then this is clearly not correct, *i.e*., it is not equivalent to a model that simultaneously applies the two models. (The impact is small, however, as it only affects sites with more than one mutation.) The solution is to make the Jukes–Cantor model *state-independent* (also called “parent-independent”), by placing mutations at rate $4/3\mu_{1}$ and then choosing the derived state for each mutation *independently* of the parent (so that one-fourth of mutations will be silent). If so—and, more generally, if the first mutational process put down is state-independent—then the result of sequentially applying the two mutation models is equivalent to the simultaneous model. To facilitate this, many mutation models have a state_independent option that increases the number of silent mutations and makes the model closer to state-independent.

Silent mutations are fully supported by tskit, which correctly accounts for their presence when computing statistics and performing other operations. For example, silent mutations have no effect on calculations of nucleotide site diversity.

### Time complexity of Hudson’s algorithm

As discussed in the *Recombination* section, the time complexity of Hudson’s algorithm is predicted to be quadratic in the population scaled recombination rate $\rho =4N_{e}L$ (where *L* is the length of the genome in units of recombination distance) by Equation (1). Figure 8 shows the running time for simulations with a variety of population sizes, chromosome length, and sample sizes, and shows this quadratic prediction is well supported by observations (see also Kelleher *et al.* 2016, Figure 2). We also see that the dependence on *n* is quite weak, since increasing sample size 100-fold only increases run time by a factor of 2 or so. However, the ${\;\text{log}\;}^{2}n$ factor implied by Equation (1) (the sum is a harmonic number and can be approximated by log n) is not well supported by observed run times (or numbers of events) except possibly at very large values of *ρ*. It therefore appears that a different dependence on *n* is required to accurately predict simulation time for a given *ρ* and *n*.

Figure 8 is a useful yardstick, allowing us to predict how long simulations should take for a wide range of species. Taking a typical chromosome to be 1 Morgan in length, these plots show, roughly, that simulating chromosome-length samples from a population of thousands of individuals takes seconds, while samples from a population of tens of thousands take minutes. Simulating whole chromosomes for many species is very fast, with 1000 samples of chromosome 1 for *Arabidopsis thaliana* taking less than a second, and a few minutes for dogs and humans. However, the dependence on *ρ is* quadratic, and if *ρ* is sufficiently large, simulations may not be feasible. For example, the *Drosophila melanogaster* chromosome 2 L is about 23.5 Mb long with an average recombination rate of around $2.4\times 10^{-8}$, so L≈0.57, and with $N_{e}=1.7\times 10^{6}$ (Li and Stephan 2006), $N_{e}L\approx 10^{6}$, so extrapolating the curve in Figure 8B predicts that simulation would require around 177 h for 1000 samples. For such large values of *ρ*, we recommend users consider approximate simulations. Since msprime does not currently have efficient implementations of approximate coalescent with recombination models, in these cases, we recommend using SMC-based methods such as scrm, particularly if sample sizes are small. In practice, to predict the running time of a given simulation in msprime, we recommend that users measure run time in a series of simulations with short genome lengths and the desired sample size, and then predict run time by fitting a quadratic curve to genome length as in Figure 8. It is important to note that the quadratic curves in the two panels of Figure 8 are different, and predicting the run times of days-long simulations using the timing of seconds-long runs is unlikely to be very accurate.

What about simulations with changing population size? To understand how run time depends on demography, it helps to consider why run time is quadratic in *ρ*. At any point in time, msprime must keep track of some number of lineages, each of which contains some number of chunks of genetic material. Common ancestor events reduce the number of lineages, and recombination events increase their number. However, with long genomes, only a small fraction of the common ancestor events will involve overlapping segments of ancestry and lead to coalescence in the marginal trees. Such disjoint segments are often far apart (on average, about distance L/2), and so recombine apart again immediately; it is these large numbers of rapid and inconsequential events that lead to the quadratic run time. The maximum number of lineages occurs when the increase and decrease in numbers of lineages due to common ancestor and recombination events balance out. To get an idea of run time we can estimate when this balance occurs. Suppose that the maximum number of lineages is *M*; at this time, the rate of common ancestor events is $M(M-1)/(4N_{e})$ and the total rate of recombination is Mℓ, where ℓ is the mean length of genome carried by each lineage (including “trapped” non-ancestral material). At the maximum, coalescence and recombination rates are equal, so a typical segment of ancestry will spend roughly half its time in a lineage with at least one other such segment—and, since such lineages carry at least two segments, at most one-third of the lineages carry long trapped segments of ancestry. Since the maximum number of lineages is reached very quickly (Nelson *et al.* 2020), this implies that ℓ≈L/6. Setting the rates of recombination and common ancestor events to be equal and solving for *M*, we find that *M* is roughly equal to $LN_{e}$. The number of lineages then decreases gradually from this maximum on the coalescent time scale, and therefore over roughly $2N_{e}$ generations. Since the total rate of events when the maximum number of lineages is present is roughly $L^{2}N_{e}/6$, then the total number of events is proportional to ${\left(LN_{e}\right)}^{2}$—*i.e.*, proportional to $\rho^{2}$.

What does this tell us about run time for simulating time-varying population sizes? Suppose that population size today is *N*_1_, while *T* generations ago it was *N*_2_. Does the run time depend more on $4N_{1}L$ or $4N_{2}L$? The answer depends on how *T* compares to *N*_1_: if $T/N_{1}\ll 1$ then the number of extant lineages remaining after *T* generations is likely to be substantial, and the algorithm runtime is primarily determined by *N*_2_. Conversely, if $T/N_{1}\gg 1$, then few extant lineages are likely to remain by time *T* and runtime depends mainly on *N*_1_. For instance, in many agricultural species $N_{1}\approx 100$, while $N_{2}\approx 10^{5}$, and the run time will depend critically on *T*—in other words, simulation will be quick in a species with a strong domestication bottleneck, and slow otherwise.

### Selective sweeps model

Sweep trajectories are generated in msprime using a jump process approximation to the conditional diffusion of an allele bound for fixation (Coop and Griffiths 2004). The jump process moves back in time following the beneficial allele frequency, *P*, from some initial frequency (*e.g.*, *P *=* *1) back to the origination of the allele at p=1/(2N), tracking time in small increments *δt*. Then, given the frequency *p* at time *t* ago, the frequency p′ at time t+δt ago is given by

$$p\prime =\{\begin{array}{cc} p+\mu (p)\delta t+\sqrt{p(1-p)\delta t} & \text{with}\;\text{probability}\;1/2\\ p+\mu (p)\delta t-\sqrt{p(1-p)\delta t} & \text{with}\;\text{probability}\;1/2 \end{array}$$

where

$$\mu (p)=\frac{\alpha p(1-p)}{\tanh(\alpha (1-p))}.$$

Here, α=2Ns and *s* is the fitness advantage in homozygotes. This model assumes genic selection (*i.e.*, that the dominance coefficient *h *=* *0.5), but can be generalized straightforwardly to include arbitrary dominance. We can also define trajectories to model neutral alleles and soft selective sweeps, which we plan as future additions to msprime.

### Likelihood calculations

We provide two functions to facilitate likelihood-based inference. Both are implemented only for the simplest case of the standard ARG with a constant population size, and require tree sequences compatible with the record_full_arg option as their arguments.

The msprime.log_arg_likelihood (ts, r, N) function returns the natural logarithm of the sampling probability of the tree sequence ts under the ARG with per-link, per-generation recombination probability r and population size *N* [*e.g.*, Kuhner *et al.* 2000, Equation (1)]. Specifically, the function returns the logarithm of

$${\left(\frac{1}{2N}\right)}^{q_{c}}(\prod_{i:R}rg_{i})\;\text{exp}(-\sum_{i=1}^{q}[\frac{1}{2N}\left(\begin{array}{c} k_{i}\\ 2 \end{array}\right)+rl_{i}]t_{i}),$$

where *t_i_* is the number of generations between the (i−1)th and *i*th event, *k_i_* is the number of extant ancestors in that interval, *l_i_* is the number of links in that interval that would split ancestral material should they recombine, *q* is the total number of events in the tree sequence ts, *q_c_* is the number of coalescences, R is the set of indices of time intervals which end in a recombination, and *g_i_* is the corresponding *gap*: the length of contiguous non-ancestral material around the link at which the recombination in question took place. The gap indicates the number of links (or length of genome in a continuous model) at which a recombination would result in exactly the observed pattern of ancestral material in the ARG. For a continuous model of the genome and a recombination in ancestral material, we set *g_i_* = 1 and interpret the result as a density.

The msprime.unnormalised_log_mutation_likelihood (ts, m) function returns the natural logarithm of the probability of the mutations recorded in the tree sequence ts given the corresponding ancestry, assuming the infinite sites model, up to a normalizing constant which depends on the pattern of mutations, but not on the tree sequence or the per-site, per-generation mutation probability m. Specifically, the function returns the logarithm of

$$e^{-Tm/2}\frac{{\left(Tm/2\right)}^{M}}{M!}\prod_{\gamma \in M}\frac{h_{\gamma }}{T},$$

where *T* and M are the total branch length and set of mutations in ts, respectively, *M* is the number of mutations, and for a mutation *γ*, $h_{\gamma }$ is the total branch length on which *γ* could have arisen while appearing on all of the leaves of ts it does, and on no others. Unary nodes on marginal trees arising from the record_full_arg option mean that, in general $h_{\gamma }$ corresponds to the length of one or more edges.

### Multiple merger coalescent model

Multiple merger coalescents, in which no more than one group of a random number of ancestral lineages may merge into a common ancestor at a given time, are referred to as Λ-coalescents. The rate at which a given group of *k* out of a total of *b* lineages merges is

(2) $$\lambda_{b,k}=\int_{0}^{1}x^{k-2}{\left(1-x\right)}^{b-k}\Lambda (dx)+a{𝟈}_{\left\{k=2\right\}},\;2\leq k\leq b,$$

where ${𝟈}_{\left\{A\right\}}:=1$ if *A* holds, and zero otherwise, a≥0 is a constant, and Λ is a finite measure on the unit interval without an atom at zero (Donnelly and Kurtz 1999; Pitman 1999; Sagitov 1999). There is also a larger class of simultaneous multiple merger coalescents involving simultaneous mergers of distinct groups of lineages into several common ancestors (Schweinsberg 2000). These are commonly referred to as Ξ-coalescents, and often arise from population models incorporating diploidy or more general polyploidy (Birkner *et al.* 2013a; Blath et al. 2016). To describe a general Ξ-coalescent, let Δ denote the infinite simplex

$$\Delta :=\{(x_{1},x_{2},\ldots ):x_{1}\geq x_{2}\geq \cdots \geq 0,\sum_{j=1}^{\infty }x_{j}\leq 1\}.$$

The rate of mergers is determined by $\Xi =\Xi_{0}+a\delta_{0}$, where a≥0 is a constant, *δ*_0_ is the Dirac delta measure, and $\Xi_{0}$ is a finite measure on Δ with no atom at (0, 0, …). For an initial number of blocks b≥2 and r∈{1,2,…,b−1}, let $k_{1}\geq 2,\ldots ,k_{r}\geq 2$ be the sizes of *r* merger events and $s=b-k_{1}-\cdots -k_{r}$ be the number of blocks not participating in any merger. The rate of each possible set of mergers with sizes $\left(k_{1},\ldots ,k_{r}\right)$ is

$$\begin{array}{c} \lambda_{n;k_{1},\ldots ,k_{r};s}=\int_{\Delta }\sum_{\ell =0}^{s}\sum_{i_{1},\ldots ,i_{r+\ell }=1}^{\infty }\left(\begin{array}{c} s\\ \ell \end{array}\right)x_{i_{1}}^{k_{1}}\cdots x_{i_{r}}^{k_{r}}x_{i_{r+1}}\cdots x_{i_{r+\ell }}\\ \text{all}\;\text{distinct}\\ \times {\left(1-\sum_{j=1}^{\infty }x_{j}\right)}^{s-\ell }\frac{1}{\sum_{j=1}^{\infty }x_{j}^{2}}\Xi_{0}(dx)\\ +a{𝟈}_{\left\{r=1,k_{1}=2\right\}}, \end{array}$$

and the number of such $\left(k_{1},\ldots ,k_{r}\right)$ mergers is

$$N(b;k_{1},\ldots ,k_{r})=\left(\begin{array}{c} b\\ k_{1}\ldots k_{r}s \end{array}\right)\frac{1}{\prod_{j=2}^{b}\ell_{j}!},$$

where $\ell_{j}:=\#\{i\in \{1,\ldots ,r\}:k_{i}=j\}$ is the number of mergers of size j≥2 (Schweinsberg 2000).

Viewing coalescent processes strictly as mathematical objects, it is clear that the class of Ξ-coalescents contains Λ-coalescents as a specific example in which at most one group of lineages can merge at each time, and the class of Λ-coalescents contain the Kingman-coalescent as a special case. However, viewed as limits of ancestral processes derived from specific population models, they are not nested. For example, one can obtain Λ-coalescents from haploid population models incorporating sweepstakes reproduction and high fecundity, and Ξ-coalescents for the same models for diploid populations (Birkner *et al.* 2013a). One should therefore apply the models as appropriate, *i.e*., Λ-coalescents to haploid (*e.g*., mtDNA) data, and Ξ-coalescents to diploid or polyploid (*e.g*., autosomal) data (Blath *et al.* 2016).

In msprime, we have incorporated two examples of multiple-merger coalescents. One is a diploid extension (Birkner *et al.* 2013a) of the haploid Moran model adapted to sweepstakes reproduction considered by Eldon and Wakeley (2006). Let *N* denote the population size, and take ψ∈(0,1] to be fixed. In every generation, with probability $1-\varepsilon_{N}$, a single individual (picked uniformly at random) perishes. With probability $\varepsilon_{N},\;\left\lfloor \psi N\right\rfloor$ individuals picked uniformly without replacement perish instead. In either case, a surviving individual picked uniformly at random produces enough offspring to restore the population size back to *N*. Taking $\varepsilon_{N}=1/N^{\gamma }$ for some γ>0, Eldon and Wakeley (2006) obtain Λ-coalescents for which the Λ measure in Equation (2) is a point mass at *ψ*. The simplicity of this model does allow one to obtain some explicit mathematical results (see *e.g*., Der *et al.* 2012; Eldon and Freund 2018; Freund 2020; Matuszewski *et al.* 2018), and the model has also been used to simulate gene genealogies within phylogenies (Zhu *et al.* 2015). As well as the haploid model of Eldon and Wakeley (2006), msprime provides the diploid version of Birkner *et al.* (2013a), in which individuals perish as above, but replacements are generated by sampling a single pair of diploid individuals as parents, with children sampling one chromosome from each parent. Hence, there are four parent chromosomes involved in each reproduction event, which can lead to up to four simultaneous mergers, giving rise to a Ξ-coalescent with merger rate

(3) $$\begin{array}{c} \lambda_{b;k_{1},\ldots ,k_{r};s}^{\text{Dirac}}=\frac{c\psi^{2}/4}{1+c\psi^{2}/4}\frac{4}{\psi^{2}}\sum_{\ell =0}^{s\wedge (4-r)}\left(\begin{array}{c} s\\ \ell \end{array}\right){\left(4\right)}_{r+\ell }{\left(1-\psi \right)}^{s-\ell }\\ \;\;\;\;\times {\left(\frac{\psi }{4}\right)}^{k_{1}+\cdots +k_{r}+\ell }+\frac{{𝟈}_{\left\{r=1,k_{1}=2\right\}}}{1+c\psi^{2}/4}, \end{array}$$

The interpretation of Equation (3) is that “small” reproduction events in which two lineages merge occur at rate $1/(1+c\psi^{2}/4)$, while large reproduction events with the potential to result in simultaneous multiple mergers occur at rate $\left(c\psi^{2}/4)/(1+c\psi^{2}/4\right)$.

The other multiple merger coalescent model incorporated in msprime is the haploid population model considered by Schweinsberg (2003), as well as its diploid extension (Birkner *et al.* 2018). In the haploid version, in each generation of fixed size *N*, individuals produce random numbers of juveniles $\left(X_{1},\ldots ,X_{N}\right)$ independently, each distributed according to a stable law satisfying

(4) $$\underset{k\to \infty }{\lim}Ck^{\alpha }P\left(X\geq k\right)=1$$

with index α>0, and where *C *>* *0 is a normalizing constant. If the total number of juveniles $S_{N}:=X_{1}+\cdots +X_{N}$ produced in this way is at least *N*, then *N* juveniles are sampled uniformly at random without replacement to form the next generation. As long as $E\left[X_{1}\right]>1$, one can show that $\left\{S_{N}<N\right\}$ has exponentially small probability in *N*, and does not affect the resulting coalescent as N→∞ (Schweinsberg 2003). If α≥2, the ancestral process converges to the Kingman-coalescent; if 1≤α<2, the ancestral process converges to a Λ-coalescent with Λ in Equation (2) given by the Beta(2−α,α) distribution, *i.e*.,

(5) $$\Lambda (dx)={𝟈}_{\left\{0<x\leq 1\right\}}\frac{1}{B(2-\alpha ,\alpha )}x^{1-\alpha }{\left(1-x\right)}^{\alpha -1}dx,$$

where B(a,b)=Γ(a)Γ(b)/Γ(a+b) for a,b>0 is the beta function (Schweinsberg 2003). This model has been adapted to diploid populations by Birkner *et al.* (2018), and the resulting coalescent is Ξ-coalescent with merger rate

(6) $$\lambda_{b;k_{1},\ldots ,k_{r};s}^{\text{Beta}}=\sum_{\ell =0}^{s\wedge (4-r)}\left(\begin{array}{c} s\\ \ell \end{array}\right)\frac{{\left(4\right)}_{r+\ell }}{4^{k+\ell }}\frac{B(k+\ell -\alpha ,s-\ell +\alpha )}{B(2-\alpha ,\alpha )},$$

where $k:=k_{1}+\cdots +k_{r}$ (Blath *et al.* 2016; Birkner *et al.* 2018). The interpretation of Equation (6) is that the random number of lineages participating in a potential merger is governed by the Λ-coalescent with rate Equation (5), and all participating lineages are randomly allocated into one of four groups corresponding to the four parental chromosomes, giving rise to up to four simultaneous mergers.

The stable law Equation (4) assumes that individuals can produce arbitrarily large numbers of juveniles. Since juveniles are at least fertilized eggs, it may be desirable to suppose that the number of juveniles surviving to reproductive maturity cannot be arbitrarily large. Hence we also consider an adaptation of the Schweinsberg (2003) model, where the random number of juveniles has a deterministic upper bound ϕ(N), and the distribution of the number of juveniles produced by a given parent (or pair of parents in the diploid case) is

(7) $$P\left(X=k\right)=1_{\left\{1\leq k\leq \phi (N)\right\}}\frac{\phi {\left(N+1\right)}^{\alpha }}{\phi {\left(N+1\right)}^{\alpha }-1}\left(\frac{1}{k^{\alpha }}-\frac{1}{{\left(k+1\right)}^{\alpha }}\right).$$

See Eldon and Stephan (2018) for a related model. One can follow the calculations of Schweinsberg (2003) or Birkner *et al.* (2018) to show that if 1<α<2 then, recalling that $k=k_{1}+\cdots +k_{r}$, the merger rate is

(8) $$\lambda_{b;k_{1},\ldots ,k_{r};s}^{\text{Beta},M}=\sum_{\ell =0}^{s\wedge (4-r)}\left(\begin{array}{c} s\\ \ell \end{array}\right)\frac{{\left(4\right)}_{r+\ell }}{4^{k+\ell }}\frac{B(M;k+\ell -\alpha ,s-\ell +\alpha )}{B(M;2-\alpha ,\alpha )}$$

where $B(z;a,b):=\int_{0}^{z}t^{a-1}{\left(1-t\right)}^{b-1}dt$ for a,b>0 and 0<z≤1 is the incomplete beta function, and

$$M:=\underset{N\to \infty }{\lim}\frac{\phi (N)/N}{\phi (N)/N+E\left[X_{1}\right]}\in (0,1]$$

(Chetwynd-Diggle *et al.* 2022). In other words, the measure Λ driving the multiple mergers is of the same form as in Equation (5) with 0<x≤M in the case 1<α<2 and $\underset{N\to \infty }{\lim}\phi (N)/N>0$. If α≥2 or ϕ(N)/N→0, then the ancestral process converges to the Kingman-coalescent (Chetwynd-Diggle *et al.* 2022).
